# α-Melanocyte-stimulating hormone ameliorates ocular surface dysfunctions and lesions in a scopolamine-induced dry eye model via PKA-CREB and MEK-Erk pathways

**DOI:** 10.1038/srep18619

**Published:** 2015-12-21

**Authors:** Yusha Ru, Yue Huang, Huijuan Liu, Juan Du, Zhu Meng, Zexia Dou, Xun Liu, Rui Hua Wei, Yan Zhang, Shaozhen Zhao

**Affiliations:** 1 Tianjin Medical University Eye Hospital, Tianjin Medical University Eye Institute, College of Optometry and Ophthalmology, Tianjin Medical University, Tianjin, 300384, China

## Abstract

Dry eye is a highly prevalent, chronic, and multifactorial disease that compromises quality of life and generates socioeconomic burdens. The pathogenic factors of dry eye disease (DED) include tear secretion abnormalities, tear film instability, and ocular surface inflammation. An effective intervention targeting the pathogenic factors is needed to control this disease. Here we applied α-Melanocyte-stimulating hormone (α-MSH) twice a day to the ocular surface of a scopolamine-induced dry eye rat model. The results showed that α-MSH at different doses ameliorated tear secretion, tear film stability, and corneal integrity, and corrected overexpression of proinflammatory factors, TNF-α, IL-1β, and IFN-γ, in ocular surface of the dry eye rats. Moreover, α-MSH, at 10^−4^ μg/μl, maintained corneal morphology, inhibited apoptosis, and restored the number and size of conjunctival goblet cells in the dry eye rats. Mechanistically, α-MSH activated both PKA-CREB and MEK-Erk pathways in the dry eye corneas and conjunctivas; pharmacological blockade of either pathway abolished α-MSH’s protective effects, suggesting that both pathways are necessary for α-MSH’s protection under dry eye condition. The peliotropic protective functions and explicit signaling mechanism of α-MSH warrant translation of the α-MSH-containing eye drop into a novel and effective intervention to DED.

Dry eye is a chronic and multifactorial disease with the manifestations of ocular surface discomfort and irritation and visual disturbance^1^. As its name indicates, dry eye disease (DED) is caused by insufficient protein-containing tear secretion or excessive aqueous evaporation. Either dysfunction can lead to tear hyperosmolarity and tear film instability, thereby causing damage and inflammation on ocular surface^1^,^2^. Inflammation is another pathogenic factor to the DED^3^,^4^. Both subclinical proinflammatory microenvironment and symptomatic inflammatory cell infiltration and microbial infection result in apoptosis and loss of structural integrity on ocular surface, exacerbating the dry eye condition^5^. Dry eye is highly prevalent, 5–30% of the population over the age 50 are afflicted by this disease^1^. Moreover, due to the intensive usage of electronic devises^6^ and increased popularity of refractive surgery^7^, the prevalence of DED in younger people is dramatically increased. Dry eye considerably deteriorates productivity and quality of life, and can be debilitating or even blinding if not properly treated^8^,^9^. Unfortunately, the current therapeutic modalities, such as artificial tear fluid, nonsteroidal or corticosteroidal anti-inflammatory agents, and punctual duct occlusion, are to relieve symptoms, and long-term administration of these modalities is either associated with unsatisfactory efficacy or avoided due to severe complications^10^. Therefore, a novel, effective, and safe interventional modality targeting the pathogenic factors of dry eye is urgently needed.

α-Melanocyte-stimulating hormone (α-MSH) is a 13-amino acid peptide derived from proteolysis of Proopiomelanocortin^11^. This peptide is widely expressed in the tissues of body surface, such as skin^12^ and cornea^13^. The receptors of α-MSH, melanocortin receptors, include 5 subtypes (MC1R-MC5R) and belong to the G protein-coupled receptor (GPCR) family^14^. α-MSH binds to the MCRs to mediate physiological functions, including metabolic regulation^15^ and neuroprotection^16^,^17^ in the brain. In the eye, the MCRs are widely distributed in the ocular surface tissues, including, but not limited to, the acinar cells in extraorbital and intraorbital lachrimal glands^18^. α-MSH binds to these receptors with specificity and affinity^18^,^19^, and promotes the protein secretion from the *in vitro* lacrimal gland preparation^18^. Moreover, α-MSH suppresses inflammation, inhibits the up-regulation of proinflammatory factors, and maintains retinal architecture in the uveitis induced by bacterial endotoxin^20^ and autoimmune T cell transfer^21^, respectively. Finally, α-MSH protects photoreceptors from degeneration in a rat model of retinal dystrophy^22^. We also have shown that intravitreal injections of α-MSH protect neuroretina and retinal vessels from apoptotic cell death in a rat model of streptozotocin-induced diabetes^23^. These studies indicate that α-MSH may have the potential to antagonize the pathogenic factors of the DED, particularly the aqueous-deficient subtype of the disease, without incurring undesired complications, thus it would be interesting to examine the protective effects of this peptide in a dry eye model.

We then seek to investigate the signaling pathways underlying α-MSH’s protective effects. One of the classical pathways elicited by the α-MSH-MCR system is to act through the MCR-coupling Gs protein and elevate intracellular cAMP levels, which, in turn, activate PKA-CREB pathway^24^. The increased cAMP levels have been shown to potentiate the protein secretion from the isolated lacrimal gland acini^25^,^26^. On the other hand, MEK-Erk signaling pathway is involved in anti-inflammatory and anti-apoptotic effects of α-MSH in a rat model of prolonged myocardial ischemia and reperfusion^27^; MEK-Erk1 or 2 pathway also mediates the interaction between the signaling pathways induced by α-MSH and leptin in both human embryonic kidney cells and brain microvessel endothelial cells^24^. Therefore, we hypothesize that α-MSH may exert its protective effects against the pathogenic factors of dry eye through both PKA-CREB and MEK-Erk1 or 2 pathways. To test this hypothesis, we topically applied the α-MSH-containing eye drops to the corneas of a scopolamine-induced dry eye model, a rat model sharing the pathogenesis and pathologies with human aqueous-deficient DED^28^,^29^. We found that α-MSH at different doses ameliorated the clinical signs and corrected the overexpression of proinflammatory factors in the corneas and conjunctivas of the scopolamine-induced dry eye rats. In addition, α-MSH, at the optimal dosage, exerted morphology-maintaining, anti-apoptotic, and cytoprotective effects on the ocular surface of the dry eye rats. Furthermore, our results clearly showed that these previously undescribed protective effects were mediated by both PKA-CREB and MEK-Erk pathways.

## Results

### α-MSH at different doses ameliorated corneal dysfunctions in the scopolamine-induced dry eye rats

To investigate the effects of α-MSH on corneal dysfunctions in dry eye rats, the α-MSH at the doses of 10^−5^, 10^−4^, and 10^−3^ mg/ml were topically applied to the corneas of the scopolamine-induced dry eye rats on daily basis, clinical evaluations, including Schirmer’s test, tear breakup time (BUT), and corneal fluorescein staining were conducted every week.

**Figure 1:**
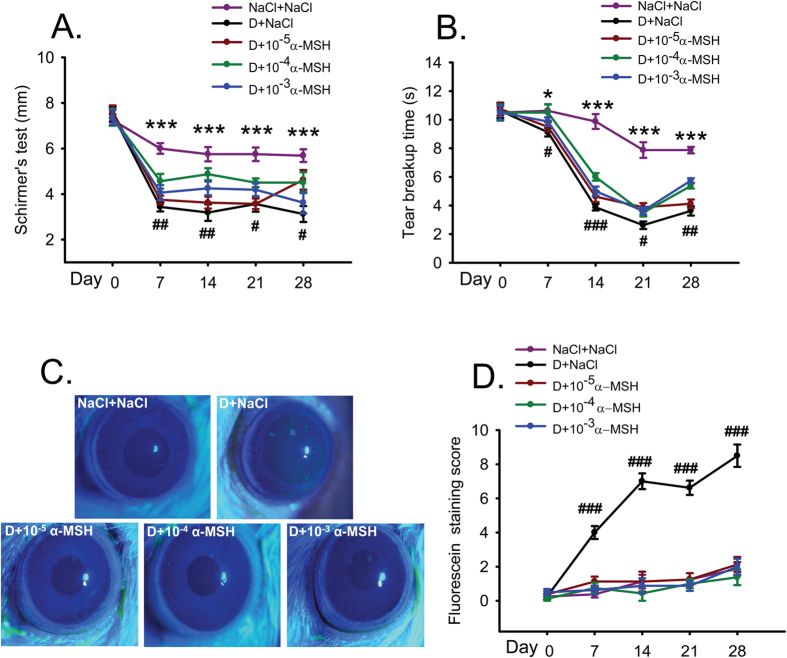
α-MSH at different doses ameliorated the ocular surface dysfunctions in the scopolamine-induced dry eye rats during the experimental time course. The animals were divided into NaCl + NaCl (purple line), D + NaCl (black line), D + 10^−5^ α-MSH (dark red line), D + 10^−4^ α-MSH (green line), and D + 10^−3^ α-MSH (blue line) groups. The Schirmer’s test (**A**), tear breakup time (**B**), and corneal fluorescein staining (**C**,**D**) were examined weekly. Representative pictures of corneal fluorescein staining were shown (**C**). The staining scores were compared (**D**). n = 8 per group. *p < 0.05, ***p < 0.001; ^#^*p* < 0.05, ^##^*p* < 0.01, ^###^*p* < 0.001. D = dry eye, NaCl = saline.

For the Schirmer’s test, saline control rats showed a steady level of tear secretion throughout the experiment except a modest 17.2% reduction on Day 7 (Fig. 1A). In contrast, the saline-treated dry eye rats exhibited a dramatic 54.2% reduction in tear secretion on Day 7. The tear secretion of the dry eye rats remained at the reduced levels, and was significantly lower than the saline controls at each time point from Day 7 to 28 (Fig. 1A, all *p* < 0.001, D+NaCl vs NaCl+NaCl). The α-MSH at 10^−4^ mg/ml significantly increased tear secretion of the dry eye rats from Day 7 to 28 (Fig. 1A, D+10^−4^ α-MSH vs D+NaCl, *p* < 0.01 for Days 7 and 14, *p* < 0.05 for Days 21 and 28). The tear secretion in 10^−5^ mg/ml α-MSH-treated dry eye group was similar to that in the saline-treated dry eye group except a significant elevation on Day 28 (Fig. 1A, *p* < 0.05, D+10^−5^ α-MSH vs D+NaCl). The alleviating effects of 10^−3^ mg/ml α-MSH on tear secretion in the dry eye rats were intermediate (Fig. 1A). The results of the Schirmer’s test suggest that α-MSH at different doses ameliorates the scopolamine-induced tear reduction, with α-MSH at 10^−4^ mg/ml being the optimal dosage.

For the BUT test, the saline controls showed a gradual diminishment from Day 0 to 21, the BUT on Day 28 in this group was similar to that on Day 21. The saline-treated dry eye rats exhibited a 14.1% reduction in the BUT in the 1^st^ week, followed by a plummet of more than 60% during the following weeks. The BUT in the saline-treated dry eye rats was significantly less than the saline controls (Fig. 1B, D+NaCl vs NaCl+NaCl, *p* < 0.05 for Day 7, *p* < 0.001 for Days 14, 21, and 28). α-MSH of different doses improved the BUT in the dry eye rats. The BUT of 10^−4^ mg/ml α-MSH-treated dry eye rats at each time point, albeit at the subnormal level, was significantly greater than that in the saline-treated counterparts (Fig. 1B, D+10^−4^ α-MSH vs D+NaCl, *p* < 0.05 for Days 7 and 21, *p* < 0.001 for Day 14, *p* < 0.01 for Day 28).

The corneas of saline control rats were transparent, smooth, and without fluorescein staining. The saline-treated dry eye rats exhibited the punctate or patchy staining, and even occasional plaques on the corneas, accordingly, the staining scores in this group were significantly higher than the saline controls (Fig. 1C,D, all *p* < 0.001, D+NaCl vs NaCl+NaCl). Notably, α-MSH, at all the doses, substantially alleviated corneal damage in the dry eye rats, with the fluorescein staining scores being similar to the saline controls (Fig. 1C,D, all *p* > 0.05, NaCl+NaCl vs D+10^−5^ α-MSH, NaCl+NaCl vs D+10^−4^ α-MSH, NaCl+NaCl vs D+10^−3^ α-MSH). These results suggest a strong protection of α-MSH against the scopolamine-induced corneal epithelial damage and tight junction disruption.

### α-MSH at different doses corrected overexpression of proinflammatory factors in the ocular surface of the dry eye rats

**Figure 2:**
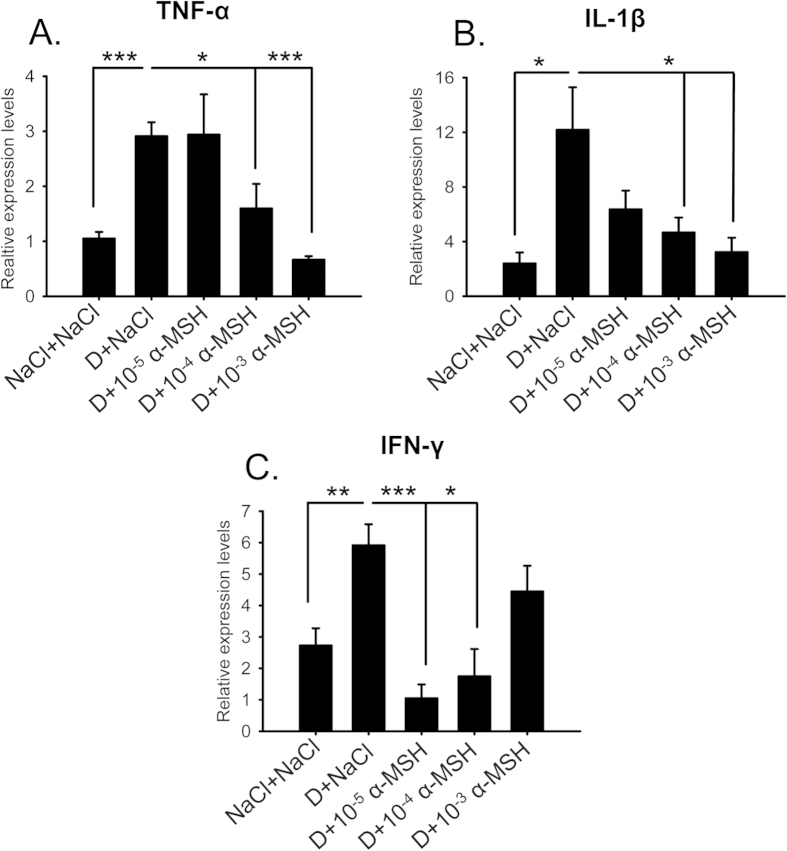
α-MSH at different doses corrected the overexpression of proinflammatory factors in the ocular surface of the dry eye rats at the end of the experiment. The animals were divided into NaCl + NaCl, D + NaCl, D + 10^−5^ α-MSH, D + 10^−4^ α-MSH, and D + 10^−3^ α-MSH groups. On day 28, the relative expression levels of the proinflammatory factors, including TNF-α (**A**), IL-1β (**B**), and IFN-γ (**C**) in the corneas and conjunctivas were measured by qPCR. n = 6 per group. *p < 0.05, **p < 0.01, ***p < 0.001. D = dry eye, NaCl = saline.

To study the protection of α-MSH on the ocular surface of the dry eye rats at the molecular level, the relative expression levels of proinflammatory factors were examined on Day 28, the end time point of dry eye induction and α-MSH intervention. The quantitative RT-PCR (qPCR) results showed the significant up-regulation in TNF-α, IL-1β, and IFN-γ mRNA levels in the saline-treated dry eye rats, as compared to the saline controls (Fig. 2A–C, D+NaCl vs NaCl+NaCl, *p* < 0.001 for TNF-α, *p* < 0.05 for IL-1β, *p* < 0.01 for IFN-γ). α-MSH at different doses decreased the transcript levels of TNF-α and IL-1β (Fig. 2A,B), and increased the levels of IFN-γ (Fig. 2C) in the corneas and conjunctivas of the dry eye rats, suggesting an improvement on the microenvironment on ocular surface by this peptide. Furthermore, α-MSH at 10^−4^ mg/ml is the only dose that renders the expression levels of the 3 proinflammatory factors similar to the saline controls (Fig. 2A–C, NaCl+NaCl vs D+10^−4^ α-MSH, *p* = 0.228 for TNF-α; *p* = 0.142 for IL-1β; *p* = 0.345 for IFN-γ). Taken together, α-MSH at 10^−4^ mg/ml significantly ameliorates the corneal dysfunctions and normalizes the expression of proinflammatory factors, therefore, is selected as the optimal dose to be used in the following experiments.

### Pharmacological blockade of PKA and Erk pathways abolished α-MSH’s amelioration on the corneal dysfunctions in the dry eye rats

**Figure 3:**
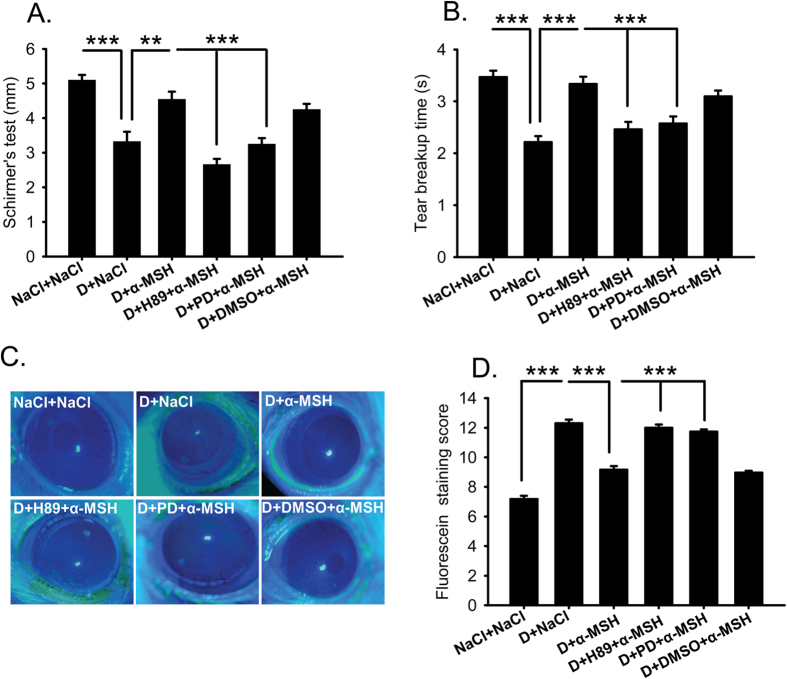
Pharmacological blockers of PKA and Erk pathways abolished α-MSH’s amelioration on corneal dysfunctions in the dry eye rats. α-MSH, at the optimal dose of 10^−4^ mg/ml, significantly improved the values of Schirmer’s test (**A**), tear breakup time (**B**), and corneal fluorescein staining (**C**,**D**) in the dry eye rats. Addition of H89 and PD98059, the pharmacological blockers to PKA and Erk pathways, respectively, abolished α-MSH’s amelioration on the corneal dysfunctions in the dry eye rats (**A–D**). The representative pictures of corneal fluorescein staining were shown (**C**). n = 16–32 per group. **p < 0.01, ***p < 0.001. D = dry eye, NaCl = saline, PD = PD98059.

To explore the signaling pathways mediating α-MSH’s protective effects on the corneas of dry eye rats, H89 and PD98059, the two widely used pharmacological blockers that block PKA^30^ and Erk^24^ pathways, respectively, were applied with 10^−4^ mg/ml α-MSH everyday, then the clinical evaluations were performed on Day 28. Consistent with the results described above, the tear secretion and BUT were significantly reduced, and the fluorescein staining scores were increased in the saline-treated dry eye rats relative to the saline controls (Fig. 3, D+NaCl vs NaCl+NaCl, all *p* < 0.001). The abnormalities were rectified by topical applications of α-MSH (Fig. 3, D+NaCl vs D+α-MSH, *p* < 0.01 for Schirmer’s test, *p* < 0.001 for BUT and fluorescein staining). Importantly, both H89 and PD98059 abrogated the α-MSH’s improvements on clinical signs in the dry eye rats (Fig. 3, D+α-MSH vs D+H89+α-MSH, D+α-MSH vs D+PD+α-MSH, all *p* < 0.001), whereas the vehicle control, Dimethyl Sulfoxide (DMSO) did not (Fig. 3, D+α-MSH vs D+DMSO+α-MSH, *p* = 0.311 for Schirmer’s test, *p* = 0.249 for BUT, *p* = 0.414 for fluorescein staining). Furthermore, the tear secretion in the H89+α-MSH-treated dry eye group was significantly lower than that in the PD98059+α-MSH-treated dry eye group (Fig. 3A, *p* < 0.05), while significant differences were not found in BUT and fluorescein staining between the two blocker-treated groups (Fig. 3B,D, *p* = 0.585 for BUT, *p* = 0.326 for fluorescein staining). These results suggest that both PKA and Erk pathways are required for α-MSH’s amelioration in the corneal dysfunctions in the scopolamine-induced dry eye rats, whereas PKA pathway is more important in mediating α-MSH’s promotion on tear secretion.

### α-MSH protected corneal morphology in the dry eye rats through PKA and Erk pathways

**Figure 4:**
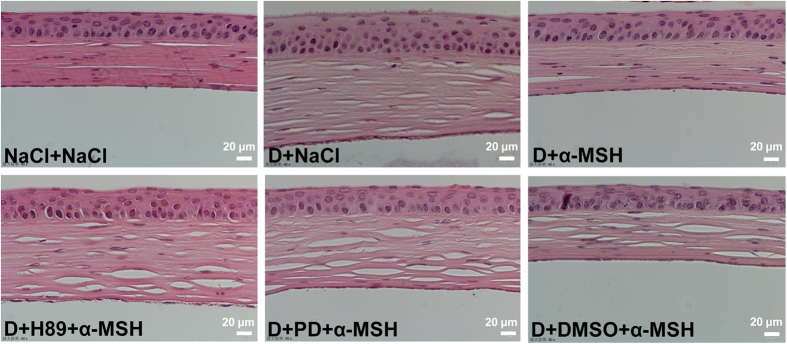
α-MSH maintained corneal morphology via PKA and Erk pathways in the dry eye rats. The animals were divided into NaCl + NaCl, D + NaCl, D + 10^−4^ α-MSH, D + H89 + 10^−4^ α-MSH, D + PD + 10^−4^ α-MSH, and D + DMSO + 10^−4^ α-MSH groups. The effects of α-MSH and the pharmacological blockers, H89 and PD98059, on corneal morphology in the dry eye rats were examined by H&E staining on Day 28. n = 8 per group. D = dry eye, NaCl = saline, PD = PD98059.

H&E staining revealed that the corneas of saline controls have smooth surface, and are composed of 4–6 layers of delicately-arrayed epithelia, among which a simple columnar epithelium was densely packed at the bottom (Fig. 4). The saline-, H89+α-MSH-, and PD98059+α-MSH-treated dry eye groups showed prominently thicker corneas with less smooth surface, increased layers of epithelia, as well as edematous basal epithelia and stroma (Fig. 4). Whereas α-MSH- and α-MSH+DMSO-treated dry eye corneas were similar to the saline controls, hyperplasia and edema were not observed in these two groups (Fig. 4). These results suggest the protective effects of α-MSH on cornea morphology in dry eye rats, and that both PKA and Erk pathways are necessary for the protective effects.

### α-MSH inhibited apoptosis in the corneas of dry eye rats via PKA and Erk pathways

**Figure 5:**
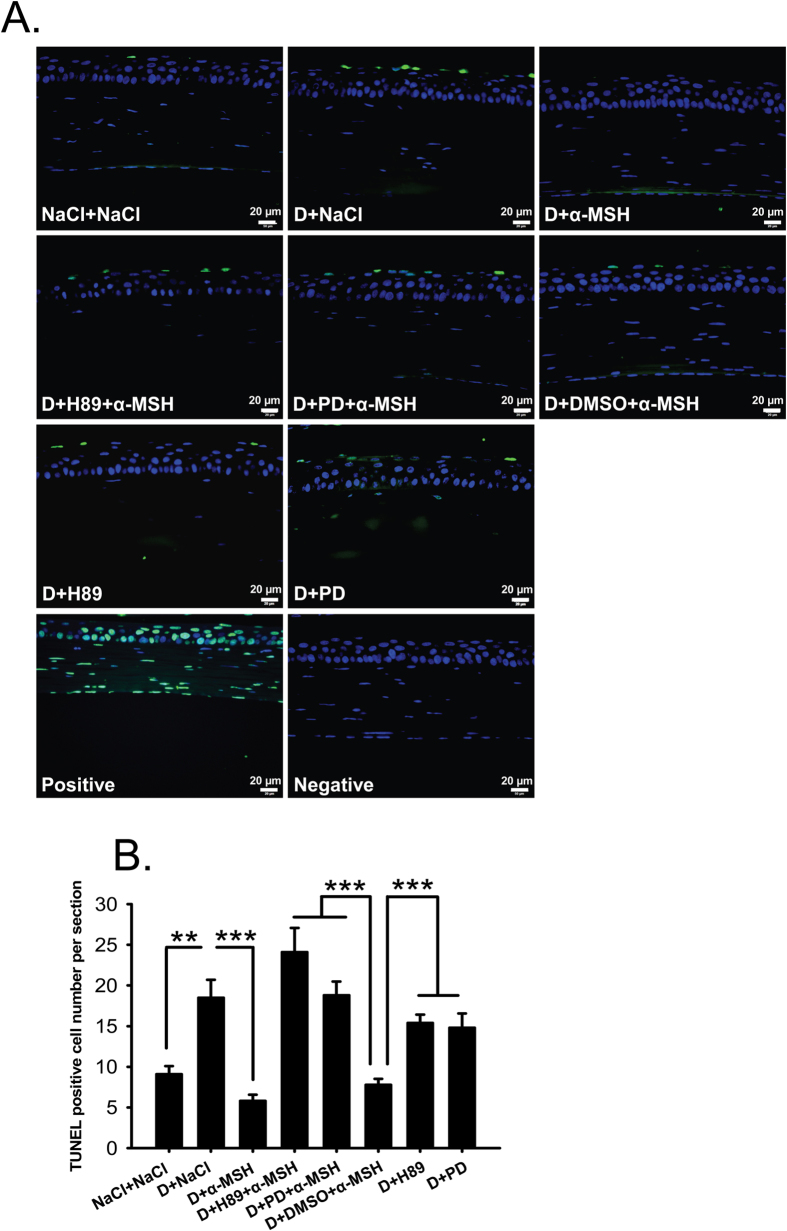
α-MSH inhibited apoptosis through PKA and Erk pathways in the dry eye corneas. The animals were divided into NaCl + NaCl, D + NaCl, D + 10^−4^ α-MSH, D + H89 + 10^−4^ α-MSH, D + PD + 10^−4^ α-MSH, D + DMSO + 10^−4^ α-MSH, D + H89, D + PD groups. The effects of α-MSH, H89, PD98059 alone or α-MSH in presence of either blocker on apoptosis in the dry eye corneas were examined by TUNEL staining on Day 28. Representative pictures of each group were shown (**A**). The estimated representation of the TUNEL-positive cell number per corneal section was compared (**B**). n = 5–10 per group. **p < 0.01, ***p < 0.001. D = dry eye, NaCl = saline, PD = PD98059.

TUNEL staining revealed a significantly higher number of TUNEL-positive cells in the corneas of saline-treated dry eye rats than the saline controls (Fig. 5B, D+NaCl vs NaCl+NaCl, *p* < 0.01). The TUNEL-positive signals were colocalized with DAPI staining, and were detected in both corneal epithelia and stroma (Fig. 5A). α-MSH substantially decreased the number of TUNEL-positive cells in the corneas of dry eye rats (Fig. 5A,B, D+NaCl vs D+α-MSH, *p* < 0.001). Whereas the TUNEL-positive cell number was significantly augmented by addition of H89 or PD98059 to the corneas of α-MSH-treated dry eye rats (Fig. 5A,B, D+α-MSH vs D+H89+α-MSH, D+α-MSH vs D+PD+α-MSH, both *p* < 0.001). The TUNEL-positive cell number in H89 and α-MSH-treated dry eye group was even greater than PD98059 and α-MSH-treated dry eye group, while the difference was not significant (Fig. 5B, D+H89+α-MSH vs D+PD+α-MSH, *p* = 0.141). H89 or PD98059 alone did not significantly change the apoptotic cell number in the corneas of dry eye rats, suggesting that these blockers do not have direct effects on corneal apoptosis under dry eye condition (Fig. 5A,B, D+H89 vs D+NaCl, *p* = 0.359; D+PD vs D+NaCl, *p* = 0.296). DMSO did not affect the anti-apoptotic effects of α-MSH either (Fig. 5A,B, D+NaCl vs D+DMSO+α-MSH, D+H89 vs D+DMSO+α-MSH, D+PD vs D+DMSO+α-MSH, *p* < 0.001; D+α-MSH vs D+DMSO+α-MSH, *p* = 0.073;). These results suggest that PKA and Erk pathways are essential to α-MSH’s anti-apoptotic effects in the corneas of dry eye rats, and the abolishment of anti-apoptosis by blockade of PKA or Erk pathway is α-MSH-dependent.

### α-MSH maintained the size and number of conjunctival goblet cells in dry eye rats via PKA and Erk pathways

**Figure 6:**
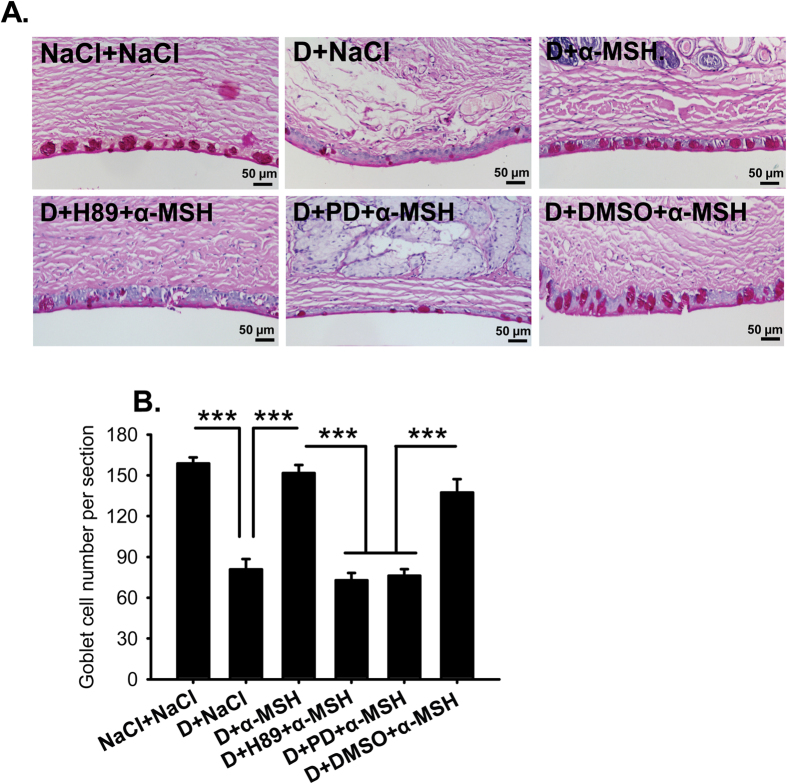
α-MSH restored the number and size of conjunctival goblet cells via PKA and Erk pathways in the dry eye rats. The rats were divided into NaCl + NaCl, D + NaCl, D + 10^−4^ α-MSH, D + H89 + 10^−4^ α-MSH, D + PD + 10^−4^ α-MSH, and D + DMSO + 10^−4^ α-MSH groups. The effects of α-MSH, and α-MSH in the presence of H89 or PD98059 on the number and size of conjunctival goblet cells in the dry eye rats were examined by PAS staining on Day 28. The representative pictures of PAS staining for each group were shown (**A**), and the estimated representation of the conjunctival goblet cell number per section was quantified (**B**). n = 10–15 per group. ***p < 0.001. D = dry eye, NaCl = saline, PD = PD98059.

Gel-forming mucins, particularly mucin 5AC (Muc5AC), are secreted by conjunctival goblet cells to attract water and stabilize tear film^31^. Because tear secretion is severely reduced in the dry eye rats, and the secreted mucins are hard to detect by the conventional immunohistochemistry, the goblet cell number revealed by Periodic acid–Schiff (PAS) staining is employed as an indicator of mucin secretion^32^. The PAS staining showed a simple columnar epithelium filled with glycoproteins aligning on the edge of conjunctiva in saline controls (Fig. 6A). In the saline-treated dry eye rats, not only the number of goblet cells was reduced 50% (Fig. 6B, NaCl+NaCl vs D+NaCl, *p* < 0.001), the size of the residual goblet cells also shrunk (Fig. 6B), indicating a dramatic reduction in mucin secretion in these animals. α-MSH restored the number and size of the goblet cells in the dry eye rats to the normal levels (Fig. 6, D+NaCl vs D+α-MSH, *p* < 0.001; D+α-MSH vs NaCl+NaCl, *p* = 0.348); whereas addition of H89 or PD98059, but not DMSO, with α-MSH abolished the protecting effects of this peptide (Fig. 6, D+H89+α-MSH vs D+α-MSH, D+PD+α-MSH vs D+α-MSH, both *p* < 0.001; D+DMSO+α-MSH vs D+α-MSH, *p* = 0.257). These results indicate that α-MSH recovers the size and number of the conjunctival goblet cells, thereby recuperating mucin secretion from these cells, in the dry eye rats via PKA and Erk pathways. These results are consistent with the ameliorating effects of α-MSH in Schirmer’s and BUT tests (Fig. 2A,B).

### α-MSH activated PKA and Erk pathways in the ocular surface of dry eye rats

**Figure 7:**
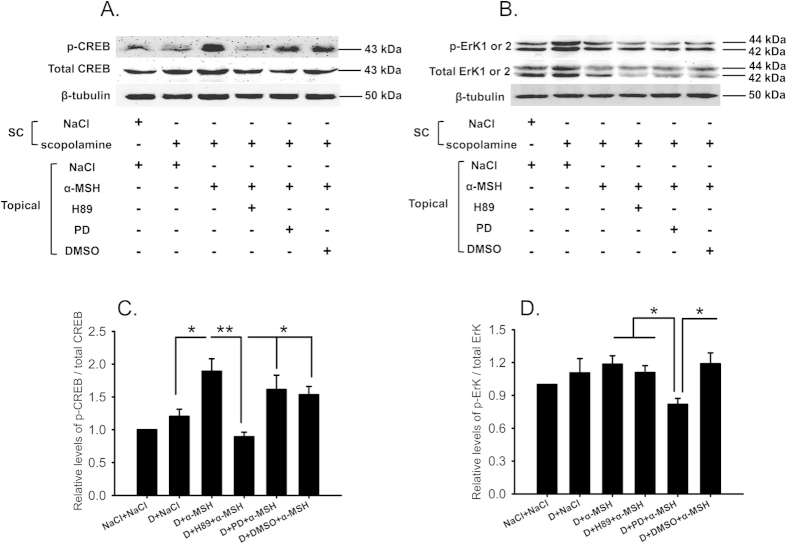
α-MSH activated PKA and Erk pathways in the ocular surface of the dry eye rats. The effects of α-MSH on PKA and Erk signaling pathways were examined by western blots. The representative blots for p-CREB and total CREB ((**A**) top panel), p-Erk1 or 2 and total Erk1 or 2 ((**B**) top panel) were shown. β-tubulin was included as the loading control. The treatments the animals had undergone were shown in the bottom panel of (A) and (B). The relative ratios of p-CREB over total CREB and p-Erk1 or 2 over total Erk1 or 2 were shown in (**C**,**D**), respectively. n = 5 per group. *p < 0.05, **p < 0.01. D = dry eye, NaCl = saline, PD = PD98059.

The expression of the signaling molecules in PKA and Erk pathways, CREB and Erk1 or 2, and their phosphorylated forms (p-CREB, p-Erk1 or 2) were examined by western blots. The specific protein bands of total CREB and p-CREB were detected with the respective antibodies at the expected molecular weight of 43 kDa (Fig. 7A). The expression levels of total CREB were not significantly altered across the board (Fig. 7A). Therefore, a dramatic induction in p-CREB by α-MSH in the dry eye corneas and conjunctivas translated into a 2-fold enhancement in the ratio of p-CREB over total CREB relative to saline controls (Fig. 7A,C, D+α-MSH vs NaCl+NaCl, *p* < 0.01). This ratio was lowered 52.8% by H89, but was not significantly affected by PD98059 or DMSO (Fig. 7A,C, D+α-MSH vs D+H89+ α-MSH, *p* < 0.01; D+α-MSH vs D+PD+α-MSH, *p* = 0.364; D+α-MSH vs D+DMSO+α-MSH, *p* = 0.234), suggesting the potent and specific inhibition of α-MSH-mediated activation of PKA pathway by H89. On the other hand, both phosphorylated and total Erk1 or 2 were detected specifically with the respective antibodies at the molecular weight of 42 and 44 kDa (Fig. 7B). Both p-Erk1 or 2 and total Erk1 or 2 were up-regulated in the saline-treated dry eye group, thereby leading to a minimal increment in the ratio of p-Erk1 or 2 over total Erk1 or 2 compared to the saline controls (Fig. 7B,D). α-MSH, however, did induce a modest and significant up-regulation in the ratio of p-Erk1 or 2 over total Erk1 or 2 in the dry eye group relative to the saline controls (Fig. 7B,D, *p* < 0.05, D+ α-MSH vs NaCl+NaCl), and this up-regulation was significantly suppressed by PD98059, but not by H89 and DMSO. (Fig. 7B,D, D+α-MSH vs D+PD+α-MSH, D+H89+α-MSH vs D+PD+α-MSH, D+DMSO+α-MSH vs D+PD+α-MSH, all *p* < 0.05). The result confirmed that PD98059 specifically inhibited the α-MSH-induced activation of Erk pathway in the corneas and conjunctivas of the dry eye rats.

## Discussion

In this study, we for the first time applied α-MSH to the ocular surface of the scopolamine-induced dry eye model, a rat model that closely resembles the clinical manifestations and pathogenesis of aqueous-deficient DED^28^,^29^. We found that topical administrations of this peptide at different doses ameliorated the corneal dysfunctions and corrected the overexpression of proinflammatory factors in the scopolamine-induced dry eye rats (Figs 1 and 2). α-MSH, at the optimal dosage of 10^−4^ mg/ml, maintained normal morphology and inhibited apoptosis in epithelium and stroma of the dry eye corneas (Figs 4 and 5). Moreover, α-MSH restored the number and size of conjunctival goblet cells in the dry eye rats (Fig. 6). As for the mechanisms, α-MSH activated PKA and Erk pathways in the dry eye ocular surface tissues (Fig. 7), and blockade of either pathway by the specific pharmacological blocker abolished the beneficial effects of α-MSH (Figs 3, 4, 5, 6), suggesting that α-MSH exerts its protection via both pathways. The striking protective effects and the clear mechanism of action of α-MSH support further developing the α-MSH-containing eye drop as a novel and effective intervention to dry eye disorder.

Subcutaneous administrations of scopolamine hydrobromide, a non-selective antagonist to muscarinic acetylcholine receptors^33^, caused dramatic and persistent reductions in Schirmer’s test value on rat corneas (Fig. 1A), suggesting the markedly reduced secretion of tears, including water, electrolytes, and proteins, from the lachrimal glands. The paucity of a protein-rich aqueous gel, accounting for 90% of tear film thickness^9^, contributed substantially to tear film instability, hence lead to a significantly shortened BUT (Fig. 1B). Topical applications of different doses of α-MSH ameliorated the values of Schirmer’s test and BUT along the experimental time course (Fig. 1A,B), the α-MSH’s improvements on tear secretion and tear film stability in the scopolamine-induced dry eye rats are consistent with its promotion on lachrimal gland secretion *in vitro*^18^,^26^. However, the alleviating effects of α-MSH on corneal dysfunctions did not correlate linearly with its concentrations, the intermediate concentration at 10^−4^ mg/ml exhibited the most remarkable improvements in corneal functions, including tear secretion and tear film stability (Fig. 1A,B). These results are consistent with our previous study on high glucose-stimulated retinal microvessel endothelial cells, where α-MSH at 0.1 μM, the intermediate concentration administered, most significantly boosted the cell viability^23^.This dose-effect characteristic was also observed in the gene expression analyses of proinflammatory factors in the current study. α-MSH at both 10^−3^ and 10^−4^ mg/ml significantly reduced TNF-α and IL-1β expression, as compared to the saline- and 10^−5^ mg/ml α-MSH-treated dry eye corneas and conjunctivas (Fig. 2A,B). However, the suppressing effects at 10^−3^ mg/ml were similar to those at 10^−4^ mg/ml (Fig. 2A,B), indicating that the highest concentration of α-MSH does not necessarily generate the optimal effects. Moreover, α-MSH at 10^−4^ and 10^−5^ mg/ml, but not at 10^−3^ mg/ml, significantly suppressed the expression of IFN-γ, and α-MSH at the lowest concentration even exhibited the most significant suppressing effects (Fig. 2C). Nevertheless, IFN-γ is required to promote the suppressive response of regulatory T cells^34^ and mucin production^35^ in ocular surface, the two important factors known to ameliorate the dry eye condition^36^,^37^. Therefore, the results of corneal functional assays and proinflammatory factor expression profiling suggest that α-MSH at the intermediate concentration, 10^−4^ mg/ml, is the optimal dosage to improve corneal functions and maintain appropriate ocular surface microenvironment.

Fluorescein staining is another method for ocular surface evaluation^32^. The staining on the corneas is due to the damage of corneal epithelia and disruption of tight junctions among these cells^38^. As expected, the corneas of saline-treated dry eye rats were diffusely and heavily stained (Fig. 1C,D). This observation in animals is confirmed at the tissue level by H&E staining, which shows coarse surface and edematous epithelia and stroma in the dry eye corneas (Fig. 4). In contrast, α-MSH, at all doses applied, abolished the fluorescein staining on the corneas of dry eye rats (Fig. 1C,D). These results are consistent with those from Pavan and colleagues^39^ showing that topical application of α-MSH promotes corneal wound healing. The fluorescein staining results also suggest the strong protective effects of α-MSH on the intercellular tight junctions and cell integrity. The α-MSH’s maintainence on junction intactness was further revealed by the absence of edema in both epithelia and stroma of the α-MSH-treated dry eye corneas (Fig. 4). Whereas the α-MSH’s protection from cell damage in dry eye corneas could be attributed to its promotion on proliferation or inhibition on apoptosis. Given the fact that epithelial cell hyperplasia was observed in the saline- but not the α-MSH-treated dry eye corneas (Fig. 4), the α-MSH’s promotion on cell proliferation appears unlikely, although Ki67 or 5-Bromo-2´-Deoxyuridine staining is needed to exclude this possibility^40^. Therefore, TUNEL staining was performed to examine the effects of α-MSH on apoptosis. The topical applications of α-MSH substantially reduced the number of TUNEL-positive cells in the dry eye corneas (Fig. 5A,B), suggesting a prominent inhibition of apoptosis by this peptide. The anti-apoptotic effects of α-MSH on dry eye corneas are in line with our previous studies. We have shown that α-MSH significantly suppresses apoptosis in the neuroretina and retinal vessels of streptozotocin-induced diabetic rats^23^, in the high glucose-treated monkey retinal vascular endothelial cells^23^, as well as in the developing chicken retina exposed to glutamate-induced excitotoxicity^41^. Together, the results of our studies indicate that α-MSH has a wide-spectrum anti-apoptotic property across the species that is, at least partially, responsible for its cytoprotective effects.

MUC5AC, a major component of gel-forming mucins, increases tear film stability and facilitates generation of a lubricated and hydrophilic corneal surface, thus is indispensible for the health of ocular surface^31^. MUC5AC is synthesized and secreted by goblet cells in the surface of fornical conjunctiva, and the MUC5AC content in the tear film is intimately related to the goblet cell number^42^. Therefore, PAS staining of conjunctival goblet cells was used as an indicator of MUC5AC content in this study. The PAS staining exhibited a 50% loss in the number and a dramatic shrinkage in the size of the conjunctival goblet cells in the saline-treated dry eye rats (Fig. 6A,B). Whereas α-MSH restored both parameters of goblet cells in the dry eye rats (Fig. 6A,B) to the normal levels, indicating the normalized MUC5AC content and the improved tear film stability under dry eye condition. The α-MSH’s protection on conjunctival goblet cells in dry eye rats contributed to the significant BUT improvements after treatment by this peptide (Figs 1B and 3B), and could be partially due to its cytoprotective and anti-apoptotic effects.

Western blots revealed a marked induction of p-CREB, and a modest activation of p-Erk in the α-MSH-treated dry eye group relative to the saline controls (Fig. 7), suggesting the activation of PKA and Erk pathways by α-MSH. The specific blockade of either pathway by a pharmacological blocker was confirmed by the western blots (Fig. 7). More importantly, blockade of PKA or Erk pathway abrogated all the protective effects of α-MSH in the dry eye rats, including improvements in ocular surface functions (Fig. 3), maintainence of morphological integrity (Fig. 4), and anti-apoptotic and cytoprotective functions (Figs 5 and 6). Together, these results explicitly suggest that α-MSH exerts its protective effects on the dry eye ocular surface through the activation of both pathways. Nonetheless, the two pathways may have differential roles in mediating the protective effects of α-MSH. The PKA-CREB pathway is α-MSH inducible, and is more important in certain protective function of α-MSH, such as promotion on tear secretion (Fig. 3A); whereas the MEK-Erk pathway could serve as a facultative pathway, the modest activation of which may permit the fulfillment of α-MSH’s protective function. Moreover, the diminished activation of one pathway following the blockade of the other (Fig. 7C,D) suggests the promoting interactions between the two pathways under the α-MSH-treated dry eye condition. However, it has been reported that elevated intracellular levels of cAMP act through inhibition of Erk pathway to potentiate protein secretion in the isolated rat lacrimal gland acinar cells^43^,^44^. It is also reported more recently that vasoactive intestinal peptide, another peptide expressing in ocular surface and acting on GPCRs, activates both cAMP-PKA-CREB and MEK-Erk pathways in the primary rat conjunctival goblet cells^45^. These reports indicate cell-type specific interactions between the two pathways activated by GPCR agonists in the ocular surface tissues, and implicate that the extent of Erk1 or 2 activation and expression may vary among different cell types. We performed an *in vivo* experiment and analyzed the Erk1 or 2 activation and expression in the tissues of cornea and conjunctiva containing multiple cell types. Therefore, the Erk1 or 2 activation and expression in different cell types may offset each other, and the modest elevation of the phosphorylated Erk1 or 2 and the reduced expression of total Erk1 or 2 observed in western blots (Fig. 7B,D) may reflect the ensemble effects of α-MSH on the ocular surface tissues. Taken together, it would be interesting in the future study to pinpoint the hierarchy of, the interactions between, and the downstream targets of the two pathways in a cell line, e.g. corneal epithelium cells, derived from the ocular surface tissue.

One of the targets downstream PKA and Erk pathways could be the proinflammatory genes. As it has been reported that α-MSH acts through PKA pathway to inhibit NFκB activation and TNF-α up-regulation in lipopolysaccharide-stimulated leukocytes^46^. Indeed, our results in this study demonstrated that α-MSH at different doses rectified the overexpression of the proinflammatory genes, TNF-α, IL-1β, and IFN-γ in the scopolamine-induced dry eye corneas and conjunctivas (Fig. 2), suggesting the potent anti-inflammatory effects of this peptide. Inflammation is both the cause and consequence of dry eye^47^,^48^. The up-regulation of proinflammatory genes in response to extrinsic and intrinsic stimuli generates a proinflammatory microenvironment that initiates cell stress and apoptosis at the beginning of dry eye. As the disease aggravates, severe cell death, tissue damage, and the substantially reduced secretion of anti-microbial proteins and tear fluid incur sterile and non-sterile inflammation, respectively. Both types of inflammation amplify and perpetuate the dry eye condition^48^. Therefore, the anti-inflammatory effects of α-MSH contribute profoundly to its morphology maintainence, anti-apoptosis, and cytoprotection effects on dry eye ocular surface. In addition, the downstream targets may also be water and ion channels, anti-apoptotic molecules, and growth factor and receptor system, the targets matching the peliotropic protective effects of α-MSH in the ocular surface of the scopolamine-induced dry eye rats.

In conclusion, we report previously undescribed protective effects of α-MSH, including amelioration of ocular surface dysfunctions, anti-inflammation, morphology maintenance, anti-apoptosis, and cytoprotection, in a rat model of scopolamine-induced dry eye syndrome. We also indentify that α-MSH exerts the protective effects via both the inducible PKA-CREB pathway and the supportive MEK-Erk pathway. The pleiotropic protective effects and explicit signaling mechanism support the translation of the α-MSH-containing eye drop into an effective and novel intervention modality to the multifactorial DED.

## Methods

### Materials

Scopolamine hydrobromide was purchased from Sigma-Aldrich (St. Louis, MO, USA), and dissolved in 0.9% sterilized NaCl solution at 6 mg/ml prior to injections. α-MSH was purchased from Calbiochem (Billerica, MA, USA), and the lyophilized powder of α-MSH was dissolved in sterilized normal saline to prepare a 3.33 mg/ml stock solution. The stock solution was aliquoted and stored at −20 °C until further usage. H89 and PD98059 were purchased from Calbiochem (Billerica, MA, USA), and dissolved in DMSO (Sigma-Aldrich, St. Louis, MO, USA) to prepare the stock solutions of 20 and 50 mM, respectively. The primary antibodies to p-CREB (#9198), CREB (#9197), p-Erk1 or 2 (#9101), Erk1 or 2 (#4695) were purchased from Cell Signaling Technology (Danvers, MA, USA). The primary antibody to β-tubulin was obtained from BD Biosciences (San Jose, CA, USA). The donkey anti-rabbit and goat anti-mouse secondary antibodies were purchased from Abcam (Cambridge, MA, USA) and Bioworld Technology (St. Louis Park, MN, USA), respectively.

### Animals

Ninety-six Wistar female rats (6 weeks of age, body weight 160–180 g) were purchased from the Chinese Academy of Military Medical Sciences (Beijing, China). The animals were maintained at 25 ± 1 °C with relative humidity of 40 ± 5% under 12 h light-dark illumination cycles (8 am to 8 pm). The animals were fed with food and water *ad lib*. All experimental procedures were approved by the Laboratory Animal Care and Use Committee of Tianjin Medical University (Permit Number: SYXK 2009-0001) and in accordance with the Association for Research in Vision and Ophthalmology Statement for the Use of Animals in Ophthalmic and Vision Research.

### Application of α-MSH at different doses to the scopolamine-induced dry eye rat model

The rats were subjected to subcutaneous (SC) injections with scopolamine hydrobromide 4 times a day (9 am, 12 am, 3 pm, and 6 pm; 0.5 ml at each time point) for 28 days. This approach has been demonstrated by us and others to successfully induce the dry eye condition in rats^28^,^29^,^49^.

The α-MSH-containing eye drop solution at 10^−3^ mg/ml was freshly prepared prior to topical applications by diluting 3 μl of the α-MSH stock solution with 10 ml sterilized normal saline. The 10^−3^ mg/ml α-MSH solution was further diluted 10- and 100-fold with the normal saline, generating the α-MSH solution at 10^−4^ and 10^−5^ mg/ml, respectively. The prepared solutions were then kept on ice until topical administration. The scopolamine-induced dry eye rats were then randomly divided into 3 groups (8 rats per group), and received topical administrations of α-MSH solution at 10^−5^, 10^−4^, and 10^−3^ mg/ml, respectively on the left eyes; whereas the right eyes of these rats were treated with sterilized 0.9% NaCl and served as vehicle controls. Both the α-MSH-containing eye drop solutions and normal saline were administered twice daily (8 am and 5 pm, the total volume 50 μl per day). Another group (8 rats) was included as saline control to receive SC injections and topical applications of sterilized normal saline with the identical volume and frequency to the dry eye groups.

### Applications of α-MSH and pharmacological blockers to the scopolamine-induced dry eye rats

In another series of experiments, the scopolamine-induced dry eye rats were randomly divided into 3 groups (16 rats per group) unless otherwise stated. These groups were topically administered on the left eyes for 28 days with the normal saline solutions containing 10^−4^ mg/ml α-MSH, 20 μM H89 and 10^−4^ mg/ml α-MSH, 50 μM PD98059 and 10^−4^ mg/ml α-MSH, and were designated as D+α-MSH, D+H89+α-MSH, and D+PD+α-MSH, respectively. The right eyes of D+α-MSH group were treated with normal saline and served as the vehicle controls for α-MSH; the right eyes of D+H89+α-MSH and D+PD+α-MSH groups were treated with the saline solution containing 10^−4^ mg/ml α-MSH and 0.1% DMSO, subserving the vehicle control for both blockers. All the topically applied solutions were administered at the same volume and frequency as described above. The group (16 rats) treated with SC injections and topical applications of normal saline was included as the saline control.

### Clinical examinations

Clinical examinations were performed on the rats at 7 am before the SC injection of scopolamine hydrobromide on Days 0, 7, 14, 21, and 28 in the standard environment (temperature 25 ± 1 °C, relative humidity 40 ± 5%) by an experienced ophthalmologist unaware of experimental grouping.

#### Schirmer’s test

The Schirmer’s test was performed as previously described^50^. Briefly, a phenol red cotton thread was placed without anesthesia in the lower fornix for 30 s at the point about one third of the distance from lateral canthus. The red portion of the thread was measured in mm.

#### BUT

Ten microliters of 0.1% liquid sodium fluorescein (Sigma-Aldrich, St. Louis, MO, USA) were instilled into the lower conjunctival fornix of the animals. After 3 artificial blinks, the cornea was observed under a slit-lamp with a cobalt blue filter (Kanghua Science & Technology, Chongqing, China). The time between the last blink and the appearance of the 1^st^ dark spot on the cornea was recorded as BUT.

#### Corneal fluorescein staining

Three minutes following the BUT test, corneal epithelium damage was examined under a slit-lamp microscope with a cobalt blue filter (Kanghua Science & Technology, Chongqing, China). The cornea was divided into 4 quadrants, and the damage in each quadrant was scored according to the criteria descried previously^51^. Briefly, no staining, 0; slightly punctate staining with less than 30 spots, 1; punctate staining with more than 30 spots, but without diffuse staining, 2; severe and diffuse staining but without positive plaque, 3; positive fluorescein plaque, 4. The scores from the 4 quadrants were summed up to represent the damage on each cornea.

#### Gene expression analyses

On Day 28, corneas and conjunctivas from saline controls, saline-treated dry eye rats, and dry eye rats treated with different doses of α-MSH (6 rats per group) were collected and frozen in liquid nitrogen. Total RNA was extracted using a GeneJET RNA Purification Kit (Thermo Fisher Scientific, Waltham, MA, USA). The concentration and purity of total RNA were examined by a Nanodrop 2000 (Thermo Fisher Scientific, Waltham, MA, USA). After digestion with DNase I, 1 μg of the total RNA was reverse transcribed using a RevertAid cDNA synthesis Kit (Thermo Fisher Scientific, Waltham, MA, USA). The expression levels of TNF-α, IFN-γ, and IL-1β genes were detected by quantitative RT-PCR (qPCR) in a HT7900 Real-Time PCR System (Applied Biosystem, Foster City, CA, USA). The cDNA content of each target gene was normalized to the internal standard GAPDH gene. The reaction mixture contains SYBR Green FastStart 2X Master Mix (Roche, Branford, CT, USA), cDNA template, and gene-specific primers (Table 1). The program was set as 2 min at 50 °C, 10 min at 95 °C, followed by 40 cycles of 15 s at 95 °C and 1 min of at 60 °C. A dissociation stage was added to check the amplicon specificity. The relative expression levels of the target genes were analyzed using a comparative threshold cycle (2^-∆∆Ct^) method.

### Histopathological examinations

On Day 28, the animals were sacrificed with excessive dose of chloral hydrate after clinical examinations. The eyeballs with intact upper and lower eyelids (8 eyes per group) were collected, post-fixed in 10% neutral buffered formalin (Sigma-Aldrich, St. Louis, MO, USA), dehydrated in graded ethanol (Sigma-Aldrich, St. Louis, MO, USA), embedded in paraffin block, and sagittally sectioned. Continuous sections (3 μm per section) of anterior segment were collected.

#### Hematoxylin and eosin staining

Ten paraffin sections from the comparable positions of the anterior segment were selected from each eye ball, and subjected to hematoxylin and eosin (H&E) staining. The stained sections were pictured under the bright field of a BX51 microscope (Olympus Optical Co. Ltd., Tokyo, Japan) with the fixed optical parameters.

#### Periodic acid–Schiff staining

Ten to fifteen paraffin sections at the matching positions of the anterior segment were stained with a Periodic acid–Schiff (PAS) Kit (Sigma-Aldrich, St. Louis, MO, USA), and counterstained with hematoxylin. The stained sections were pictured under the bright field by a BX51 microscope (Olympus Optical Co. Ltd., Tokyo, Japan) with low and high magnifications. The low magnified pictures were used to quantify the estimated representation of the conjunctival goblet cells in each section; the high magnified pictures served as the source of representative pictures.

#### Terminal deoxynucleotidyl transferase dUTP nick end labeling staining

In this experiment, the animals were grouped as described above except that the dry eye rats topically treated with H89 or PD98059 alone were added (5 rats per group). On day 28, the eye balls from each group were collected and paraffin embedded. Twelve paraffin sections from the comparable positions of the anterior segment were selected from each eye ball. Ten sections were subjected to Terminal deoxynucleotidyl transferase dUTP nick end labeling (TUNEL) staining using an *In Situ* Cell Death Detection Kit, Fluorescein (Roche, Branford, CT, USA); whereas the other two were incubated with the reaction mixture with DNase I or the mixture without terminal deoxynucleotidyl transferase, and served as the positive or negative control, respectively. After staining, the slides were mounted with ProLong Gold Antifade with DAPI reagent (Life Technologies, Grand Island, NY, USA). Pictures were taken by the cellSens Standard electronic system (Olympus Optical Co. Ltd., Tokyo, Japan) under a fluorescence microscope (BX51, Olympus Optical Co. Ltd., Tokyo, Japan). Pictures were taken with identical optical parameters at appropriate magnifications for each section. The non-overlapping low magnified pictures cover the complete corneal section, and these pictures were used to quantify the estimated representation of TUNEL-positive cells in each section. The nucleus-localized fluorescent signals with the intensity stronger than non-specific background were considered positive. The high magnification pictures served as group representatives.

### Western blot analysis

On Day 28, corneas and conjunctivas (8 rats per group) were collected, frozen in liquid nitrogen. The total proteins were extracted from the ocular surface tissue samples by a Tissue Protein Extraction Kit (CWBIO, Beijing, China), and the protein concentration determined using a Bicinchoninic Acid (BCA) Protein Assay Kit (CWBIO, Beijing, China). The western blots were conducted as preciously described^24^,^52^,^53^. Briefly, 50 μg total protein from each sample were resolved in a sodium dodecyl sulfate polyacrylamide gel, and transferred to a polyvinylidene difluoride membrane. The blots were washed, blocked with 5% non-fat dry milk, and incubated with the primary antibodies, rabbit p-CREB, CREB, p-Erk1 or 2, Erk1 or 2 (all diluted at 1:1000) at 4 °C overnight. These antibodies have been reported to reliably detect the target proteins in the literature^54^,^55^. On the next day, the blots were washed and incubated with the corresponding horseradish peroxidase-conjugated secondary antibodies at room temperature for 2 h. The protein signals were visualized with enhanced chemiluminescence plus reagents (Amersham Biosciences,, Piscataway, NJ, USA), and imaged using a Multispectral Imaging System (Biospectrum AC Chemi HR 410, UVP, LLC, Upland, CA, USA). The blots were then stripped and probed with a monoclonal antibody to β-tubulin (1:2000) to serve as an internal standard. The optical densities of the target proteins were quantified by Quantity One (Bio-Rad, Hercules, CA, USA) and normalized to that of the internal standard β-tubulin. The ratios of p-CREB over total CREB and p-Erk1 or 2 over total Erk1 or 2 were calculated, and then expressed as the fold of changes to the saline control.

### Statistical analysis

All data were expressed as Mean ± SEM. Statistical analyses were performed using Statistical Program for Social Sciences 20.0 (IBM SPSS Inc.). The data were examined by D’Agostino and Pearson omnibus normality test, those with Gaussian distribution were examined by Levene test to confirm homogeneity of variance, and then analyzed by One-way ANOVA followed by Dunnett’s post hoc; those with nonparametric distribution were analyzed by Kruskal–Wallis test followed by Dunn’s post hoc. A *p* < 0.05 was considered significant.

## Additional Information

**How to cite this article**: Ru, Y. *et al.* a-Melanocyte-stimulating hormone ameliorates ocular surface dysfunctions and lesions in a scopolamine-induced dry eye model via PKA-CREB and MEK-Erk pathways. *Sci. Rep.* **5**, 18619; doi: 10.1038/srep18619 (2015).

## Figures and Tables

**Table 1:** Primers used in qPCR.

Gene	NCBI accession #	Primer sequences
TNF-α	NM_012675.3	F: 5′-ACAAGGCTGCCCCGACTAC-3′
		R: 5′-CTCCTGGTATGAAATGGCAAATC-3′
IL-1β	E05490.1	F: 5′-AGGCTTCGAGATGAACAACAAAA-3′
		R: 5′-TCCATTGAGGTGGAGAGCTTTC-3′
INF-γ	NM_138880.2	F: 5′-CACGCCGCGTCTTGGT-3′
		R: 5′-GAGTGTGCCTTGGCAGTAACAG-3′
GAPDH	AF106860.2	F: 5′-ATGTATCCGTTGTGGATCTGACAT-3′
		R: 5′-CTCGGCCGCCTGCTT-3′
